# Evaluation of Three Medetomidine-Based Anesthetic Protocols in Free-Ranging Wild Boars (*Sus scrofa*)

**DOI:** 10.3389/fvets.2021.655345

**Published:** 2021-03-29

**Authors:** Jacopo Morelli, Sophie Rossi, Boris Fuchs, Emmanuelle Richard, Daniela S. B. Barros, Susanne Küker, Jon M. Arnemo, Alina L. Evans

**Affiliations:** ^1^Department of Forestry and Wildlife Management, Inland Norway University of Applied Sciences, Koppang, Norway; ^2^Wildlife Diseases Unit, French Agency for Biodiversity, Gap, France; ^3^François Sommer Foundation, Paris, France; ^4^Vetsuisse Faculty, Veterinary Public Health Institute, University of Bern, Bern, Switzerland; ^5^Department of Wildlife, Fish and Environmental Studies, Swedish University of Agricultural Sciences, Umeå, Sweden

**Keywords:** butorphanol, blood gas, oxygen, anesthesia, capture, medetomidine, *Sus scrofa*, wild boar (*Sus scrofa* L.)

## Abstract

Three medetomidine-based drug protocols were compared by evaluating time courses, reliability and physiological effects in wild boars. A total of 21 cage-trapped wild boars (*Sus scrofa*) were immobilized using one of the following drug combinations; MTZ: medetomidine (0.2 mg/kg) + tiletamine-zolazepam (2.0 mg/kg), MK: medetomidine (0.15 mg/kg) + ketamine (5 mg/kg), and MKB: medetomidine (0.1 mg/kg) + ketamine (5.0 mg/kg) + butorphanol (0.2 mg/kg). Induction time, recovery time, and physiological variables were recorded and arterial blood gas analysis measured twice, before and after 15 min of oxygen supplementation (0.5–1.0 L/min). For reversal, 4 mg of atipamezole per mg of medetomidine was administered intramuscularly. The boars recovered in the cage and were released once ataxia resolved. The MK group had significantly longer recovery times (mean 164 min ± 79 SD) compared to the other groups. MKB elicited longer and incomplete induction compared to the other groups (mean induction time 20 min ± 10 SD), decreasing the efficiency of the capture and increasing the risk of hyperthermia. Both ketamine-based protocols required additional ketamine intramuscularly to prolong the anesthesia after 20–40 min from induction. Agreement between the pulse oximeter and the blood gas analyzer was low, with the pulse oximeter underestimating the real values of arterial oxyhemoglobin saturation, particularly at higher readings. Mild acute respiratory acidosis (PaCO_2_ 45–60 mmHg) and mild to moderate hypoxemia (PaO_2_ 69–80 mmHg) occurred in most boars, regardless of the treatment group but especially in the MKB group. The acid-base status improved and hypoxemia resolved in all boars during oxygen supplementation, with the PaO_2_ rising above the physiological reference range (81.6–107.7 mmHg) in many individuals. MK and MKB induced safe and reliable immobilization of wild boars for at least 20 min. Supplemental oxygen delivery is recommended in order to prevent hypoxemia in wild boars immobilized with the protocols used in the present study. Long and ataxic recoveries occurred in most animals, regardless of the protocol, but especially in the MKB group.

## Introduction

Wild boars (*Sus scrofa*) are frequently captured and either physically or chemically restrained for research and management purposes (1, 2). In order to reduce the stress and safety risks for both humans and animals, chemical immobilization is the preferred method of restraint. Anesthesia is a more complex state than chemical immobilization and is defined as “drug-induced unconsciousness that is characterized by controlled but reversible depression of the central nervous system and perception. In this state, the patient is not arousable by noxious stimulation” (3). Most recommended drug protocols for wild boar anesthesia are based on one of the cyclohexamine anesthetic drug, ketamine or tiletamine (4–6). Tiletamine, which is only available in a 1:1 ratio with the benzodiazepine agonist zolazepam, is more potent and has a longer elimination half-life than ketamine. The main side effects of cyclohexamines include muscle rigidity and dose-dependent prolonged, ataxic recoveries (1, 7). By choosing a balanced anesthetic combination that includes an alpha-2 adrenoceptor agonist, such as xylazine or medetomidine, these side effects can be alleviated (4). Medetomidine is more potent than xylazine and is the preferred alpha-2 adrenoceptor agonist. Medetomidine improves muscle relaxation and potentiates the primary anesthetic drugs so that its dose can be reduced. Additionally, the effects of medetomidine can be antagonized, making the cyclohexamine combinations partly reversible. Butorphanol, an opioid, has been used as an adjunct to further improve anesthesia and reduce dose requirements of the other drugs used in a combination (8–10).

Anesthetized wild boars are prone to hyperthermia (8) and hypoxemia (11) and the body temperature and blood oxygenation of immobilized animals should be frequently monitored. Pulse-oximeters are valuable tools for assessing oxygen saturation in immobilized wildlife, especially in field settings, where blood gas analysis is not routinely conducted. Nevertheless, the tendency of pulse oximeters to either underestimate or overestimate arterial oxyhemoglobin saturation and to lack in accuracy has been previously described in several mammalian species (12–14). Such inaccuracy requires that pulse oximetry is validated with the simultaneous use of a blood gas analyzer. Most anesthetized wildlife require oxygen supplementation in order to correct hypoxemia (5, 13), which could otherwise cause multiple organ dysfunction, including but not limiting to the central nervous system (15). Ultimately, such a physiological impact might also lead to biased results of the ecological studies dependent on capturing representative animals.

The aim of the present study was to evaluate three medetomidine-based protocols for anesthesia of trapped wild boars, to assess the effects of supplemental oxygen and to evaluate the reliability of pulse oximetry in this species compared to blood gas analysis.

## Materials and Methods

### Study Area and Period

The study was conducted in the National Estate of Chambord (47°36′ N, 1°31′ E, France), a 5,439 ha enclosed park located at low elevation (72–128 meters above sea level). Wild boars are fed daily throughout the year to maintain high densities. The study was carried out in September 2015 and in April 2017, with an ambient temperature of 6.0–17.5°C and a barometric pressure (P_B_) of 754–763 mmHg (16) during the captures.

### Capture Methods and Drug Combinations

A total of 10 (2 × 1 × 1 meter) cage traps were set in the forest and baited with corn and pies daily 1 week prior the start of the captures. Traps were inspected early every morning during the capturing period. Traps were triggered by the animal when stepping on a bottom platform in the center of the trap, connected to a drop gate at the entry. In case of more than one wild boar present in the same trap, the animals were separated and moved into other cages prior to injection of the drugs. The body mass of the animals was estimated from a distance in order to adjust drug doses. The wild boars were injected with the anesthetic combination intramuscularly (IM) in the thigh muscle group using a automatic spring-loaded pole syringe including a multiuse 10 ml syringe and 30 × 2 mm needles (Dan-Inject®, Børkop DK-7080, Denmark). While the needle was changed for every injection, the syringe was not sterilized in between the injections. A total of two operators carried out the injections. Stress of the wild boars was not scored however it was higher at the time of the injection, with a remarkable stress response (i.e., charging and biting the cage). The cages were then covered with a tarp to minimize stress during induction of the drug effect.

Three different drug combinations were formulated following previous findings in similar studies in wild boar chemical immobilizations (17, 18), therefore the wild boars were divided into the following groups:

- MTZ group (medetomidine-tiletamine-zolazepam): 0.2 mg/kg of medetomidine (Zalopine®, Orion Pharma Animal Health, Turku 20101, Finland, 10 mg/ml) + 2 mg/kg of tiletamine-zolazepam (Zoletil Forte® Vet, Virbac S.A., Carros 06510, France, prepared by dissolving the powder in sterile water to a total drug concentration of 50 mg/ml).
- MK group (medetomidine-ketamine): 0.15 mg/kg of medetomidine + 5 mg/kg of ketamine (Narketan10®, Vetoquinol Ireland Ltd, Dublin 6, Ireland, 100 mg/ml).
- MKB group (medetomidine-ketamine-butorphanol): 0.1 mg/kg of medetomidine + 5 mg/kg of ketamine + 0.2 mg/kg of butorphanol (Alvegesic®, Dechra, Shrewsbury SY4 4AS, UK, 10 mg/ml).

### Monitoring and Handling

The time from the first pole syringe injection to complete immobilization (induction time) was recorded. Induction problems were defined as animal not being recumbent after 10 min or not completely immobilized 20 min post injection. Depending on animal behavior, these animals were injected with an additional half or full dose 20 min after the first injection. Once anesthesia was achieved, the animals were approached, blindfolded and placed in lateral recumbency outside the trap. Rectal temperature (Tr) was immediately measured with a digital thermometer (Wellkang Ltd, CT16 1PW, UK) placed 10 cm deep into the rectum and then monitored every 10 min. Hyperthermia was considered as at least two consecutive measures above 39.5°C. Heart rate (HR), respiratory rate (RR) and peripheral oxyhemoglobin saturation (SpO2) were recorded every 10 min from induction to recovery using a stethoscope and a a portable pulse oximeter (Nellcor® NPB-40, Nellcor Inc., Pleasanton, California, USA) with the transmissive probe placed over the lingual artery. The pulse oximeter showed the pulse amplitude at all times by means of a subjective bar graph, reflecting the quality of the reading, allowing the researchers to promptly identify the risk of inaccurate readings. The depth of the immobilization was assessed every 5 min by monitoring the palpebral and corneal reflexes, the movements of the ears, HR, RR, and occasional muscle tremor. The wild boars were classified as juveniles (<12 months of age), subadults (12–24 months of age) or adults (>24 months of age) by assessing the eruption of the incisor teeth. Body mass was measured with an analog spring scale and all captured boars were ear tagged for individual identification.

On completion of the procedures, all animals received 4 mg of atipamezole (Antisedan®, Zoetis, Parsippany NJ 07054, USA, 5 mg/ml) per mg of medetomidine in the thigh muscle group to reverse the effects of the alpha-2 adrenoceptor agonist, as recommended in previous studies (19). Time between the initial drug injection and the reversal of the anesthesia was recorded (capture duration). In case of more than one animal captured in the same trap, they were moved to individual wooden boxes to recover alone prior to be released once ataxia was no longer present. Times of first sign of recovery (head lifting), standing, walking, and leaving the site were recorded. Recovery time was defined as the time from atipamezole injection to coordinated walking or release from the individual box.

### Sample Collection and Processing

After induction of complete immobilization, an arterial blood sample was collected anaerobically from the femoral artery using heparinized 1 ml syringes (Smiths Medical ASD, Inc., Keene NH 03431, USA) and 20 × 0.6 mm needles. The sample was immediately analyzed using an i-STAT®1 Portable Clinical Analyzer and i-STAT® CG8+ and Chem8+ cartridges (Abbott Laboratories, Abbott Park IL 60064–6048, USA). The analyzer was kept in an insulated box to maintain a temperature between 16 and 30°C. Measured variables included pH, arterial partial pressure of oxygen (PaO_2_), arterial partial pressure of carbon dioxide (PaCO_2_), lactate, sodium (Na), potassium (K), chloride (Cl), ionized calcium (iCa), hematocrit (Hct), glucose and urea (BUN). PaO_2_, PaCO_2_ and pH were corrected based on Tr. Calculated values included base excess (BE), bicarbonate (${\text{HCO}}_{3}^{-}$), total carbon dioxide (TCO_2_), arterial oxyhemoglobin saturation (SaO_2_), hemoglobin (Hb), and anion gap (AG). After the arterial blood sampling, oxygen was delivered from a portable oxygen concentrator (Philips Respironics EverGo, Advanced Aeromedical, Inc., Virginia Beach VA 2345, USA) at a flow rate of 0.5–1 L/min *via* a bi-prong nasal cannula (Minton Medical Supplies PTY, Ltd., Collingwood VIC 3066, Australia) placed 10 cm deep into the nostrils. Further, a second arterial sample was taken after 15 min and processed as mentioned above to assess the effects of the supplemental oxygen. The SpO_2_ values obtained *via* pulse oximetry at the time of the arterial blood samples were recorded and any mismatch between the HR measured with the pulse oximeter and the HR counted by means of the stethoscope was ruled out, to further ensure more accurate readings from the machine. In case of a HR mismatch or a low quality of pulse detected by the pulse oximeter, the probe site was flushed with sterile crystalloid solution, wiped and the probe was reattached. The alveolar-arterial oxygen tension difference (P_(A−a)_O_2_) prior to oxygen delivery was estimated for the Tr corrected values, based on calculation of the alveolar partial pressure of oxygen (PAO_2_) calculated from the alveolar gas equation [PAO_2_ = FiO_2_ (P_B_ - P_H2O_) - (PaCO_2_/RQ)], where P_B_ = barometric pressure detected by the iSTAT® at the time of the capture, FiO_2_ = fraction of inspired oxygen (0.21) and P_H2O_ = saturated vapor pressure for water at 37°C (47 mmHg). The respiratory quotient (RQ) was assumed to be 1.1 for wild boars (2). Since the FiO_2_ was unknown at the time of the second arterial sample, P_(A−a)_O_2_ was not calculated. Hypoxemia was defined as mild (PaO_2_ 80–70 mmHg; SaO_2_ 97–90%), moderate (PaO_2_ 69–60; SaO_2_ 89–78%), or severe (PaO_2_ ≤ 59 mmHg; SaO_2_ ≤ 77%) according to the iSTAT® PaO_2_ and SaO_2_. Hypercapnia was defined as mild (PaCO_2_ 45–60 mmHg), moderate (PaCO_2_ 61–75 mmHg) and severe (PaCO_2_ > 75 mmHg) according to the iSTAT® PaCO_2_.

### Statistical Analysis

Microsoft Excel 2019 was used to conduct simple descriptive statistics including mean and standard deviation (SD) for body mass, capture parameters (drug doses, induction time, capture time, recovery time), anesthesia variables (Tr, HR, RR, SpO_2_), and all variables from blood gas, hematological, and serum biochemistry analyses, for every animal and within protocol groups. Kurtosis, skewness and their standard error were also calculated to confirm that the data within each protocol group were normally distributed.

R-3.5.3 (20) software was used to conduct a Kruskal-Wallis test by rank to investigate significant differences among protocol groups. A Dunn's test with simple Bonferroni method for *p*-value adjustment was used as *post-hoc*. Paired samples Wilcoxon test was used for the blood gas, hematological and serum biochemistry variables between the first and the second sample. Further, all simultaneous SpO_2_-SaO_2_ sets of values were matched and Lin's concordance correlation coefficient (ρ_c_) was calculated to validate the concordance between the SpO_2_ values from the pulse-oximeter to the “gold standard” SaO_2_ values from the iSTAT® analyzer, with a strong correlation considered for |ρ_c_| > 0.9. Agreement between saturation measurements was evaluated by use of Bland-Altman analysis. In this analysis, the mean of the two measurements is plotted against the difference in the measurements, and at least 95% of the elements must lay within the upper and lower limits of agreement (LoA) to suggest a fair agreement. Acceptable agreement was considered *a priori* for |LoA| < 5% between paired SaO_2_-SpO_2_ values. We will refer to this threshold as clinically acceptable limit (CAL). A Wilcoxon rank sum test was used to determine significant differences between paired SpO_2_ and SaO_2_ sets of values. Non-parametric analysis results were considered statistically significant at *p* < 0.05.

## Results

### Efficiency and Reliability of the Capture

A total of 32 animals triggered the traps during the study period: 31 wild boars, including 10 piglets which were immediately released, and a young red deer. Trapping of multiple animals in the same cage occurred in three cases and all the individuals suitable for the study were immobilized at the same time. Twenty-one wild boars were anesthetized (8 with MTZ, 7 with MK and 6 with MKB), monitored, and released in the study area. No evidence of incomplete emptying of the pole syringe was recorded. Animals in the MTZ group were younger, with a lower body mass, than those in the other two groups. Mean (± SD) body mass, drug doses, induction time, capture time, recovery time and induction problems are presented in Table 1.

**Table 1:** Drug doses and capture time course data of cage-trapped wild boars anesthetized using medetomidine-tiletamine-zolazepam (MTZ; *n* = 8) or medetomidine-ketamine (MK; *n* = 7) or medetomidine-ketamine-butorphanol (MKB; *n* = 6).

**Group**	**Sex**	**Age**		**Mass (kg)**	**M (mg/kg)**	**TZ (mg/kg)**	**K (mg/kg)**	**B (mg/kg)**	**IT (min)**	**CT (min)**	**RT (min)**	**IP (*n*)**	**A^**+**^ (*n*)**	**K^**+**^ (*n*)**
MTZ	M 5	J 2	Mean	57.6	0.28	3.06			11	66	79	2	2	0
	F 3	S 2	± SD	26.7	0.21	0.56			8	21	57			
		A 4	*n*	8	8	8			8	7	7	2	2	0
MK	M 5	J 0	Mean	74.1	0.17		5.64		11	53	164	1	1	2
	F 2	S 0	± SD	16.3	0.04		1.41		6	14	79			
		A 7	*n*	7	7		7		7	7	6	1	1	2
MKB	M 4	J 0	Mean	89.5	0.12		6.16	0.25	20	63	71	4	2	2
	F 2	S 0	± SD	21.1	0.04		2.06	0.08	10	9	51			
		A 6	*n*	6	6		6	6	6	5	4	4	2	2

*Sex (M, males; F, females), age (J, juveniles; S, subadults; A, adults), body mass, total amount of drug doses used to induce anesthesia (M, medetomidine; TZ, tiletamine-zolazepam; K, ketamine; B, butorphanol), and capture details (IT, induction time; CT, capture time; RT, recovery time; IP, induction problems; A^+^, additional 50% initial anesthetic dose; K^+^, additional ketamine administration) recorded during the anesthesia of 21 wild boars. One additional administration of 50% initial anesthetic dose IM was necessary 20 min after the first injection in one wild boar in MK group, in two wild boars in MTZ and MKB groups and are included in the drug doses reported in this table. One additional administration of 2–3 mg/kg of ketamine IM was necessary 20–40 min after the first injection to prolong the immobilization of two wild boars in MK and MKB groups and are not included in the ketamine dose reported in this table. Time variables are expressed in min (min)*.

Average initial dose of MTZ was 1.5 times higher than initially planned because lower doses were associated with induction problems (in two out of eight animals, both recumbent over 10 min after the first injection). The average actual MK and MKB doses were higher than the targeted dose by 11.7 and 12.3%, respectively, due to inaccurate body mass estimation. Overall, a second pole-syringe injection with an additional half induction dose was necessary 20 min after the first injection in 5 out of 21 boars: 2 out of 8 in the MTZ group, 1 out of 7 in the MK group and 2 out 6 in the MKB group. A higher number of induction problems occurred in the MKB group: 4 out of 6, all recumbent by 10 min from the initial injection, but not completely immobilized within 20 min. In contrast, only 1 induction problem was recorded (1 out of 7 wild boars, not fully induced by 20 min from the injection) in the MK group. No significant differences in induction times were detected among the groups.

Partial arousal during handling were more frequent in the MK (in 3 out of 7 boars) and the MKB (4 out of 6) groups than in the MTZ group (1 out of 8), requiring additional 2–3 mg/kg of ketamine IM 20–40 min from the previous full or half dose in two cases in the MK group and in other two cases in the MKB group to prolong the anesthesia.

Pre-release mortality occurred in 2 out of 32 captured animals (6.2%), with none of them being anesthetized as both deaths occurred in piglets, due to trauma by the trap closure mechanism before the inspection of the traps.

Mild to moderate ataxia was observed in all animals during the recovery period, requiring a delay in the release of animals after the antidote administration (up to 4.3 h later). Mean recovery times did not differ significantly among groups, however only 2 out of 7 individuals of the MK group were fit to be released within 1.5 h from the injection of atipamezole compared to the 6 out of 8 and the 4 out of 6 in MTZ and MKB groups, respectively. No post-release mortality was reported during the 30 days following the capture.

### Physiological Variables

**Figure 1:**
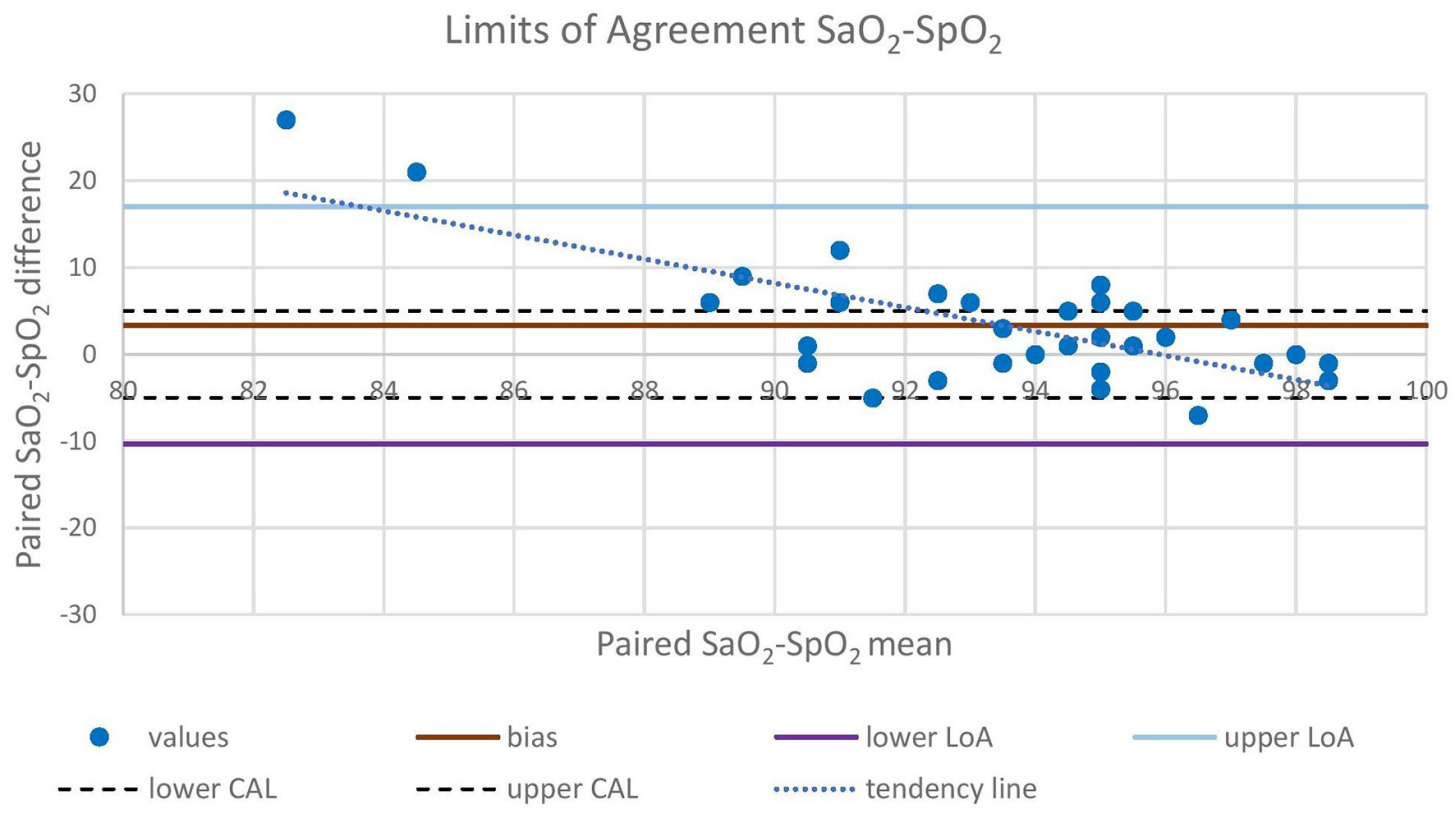
Bland-Altman analysis plots the difference between concurrent SaO_2_ and SpO_2_ values (Y-axis) against the mean between concurrent SaO_2_ and SpO_2_ values (X-axis). Mean difference (bias), upper LoA (bias + 1.96 SD), and lower LoA (bias – 1.96 SD) between paired values are represented with straight lines. Upper CAL (5%) and lower CAL (−5%) are represented with dashed lines. LoA, Limit of Agreement; CAL, Clinically Acceptable Limit; SaO_2_, arterial oxyhemoglobin saturation; SpO_2_, peripheral oxyhemoglobin saturation.

**Figure 2:**
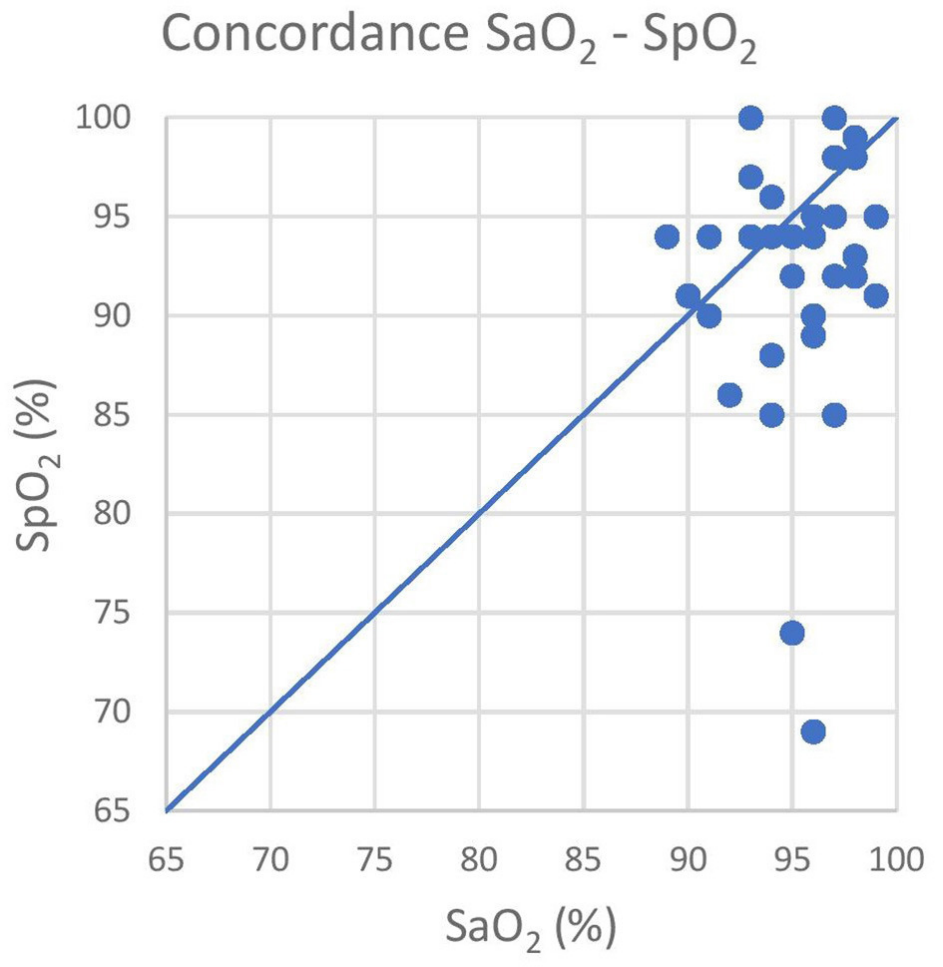
The graph shows the poor concordance between the pulse-oximeter (Y-axis) and the iSTAT (X-axis) results. The blue line represents the equality line, that is for the absolute concordance. SaO_2_, arterial oxyhemoglobin saturation; SpO_2_, peripheral oxyhemoglobin saturation.

**Figure 3:**
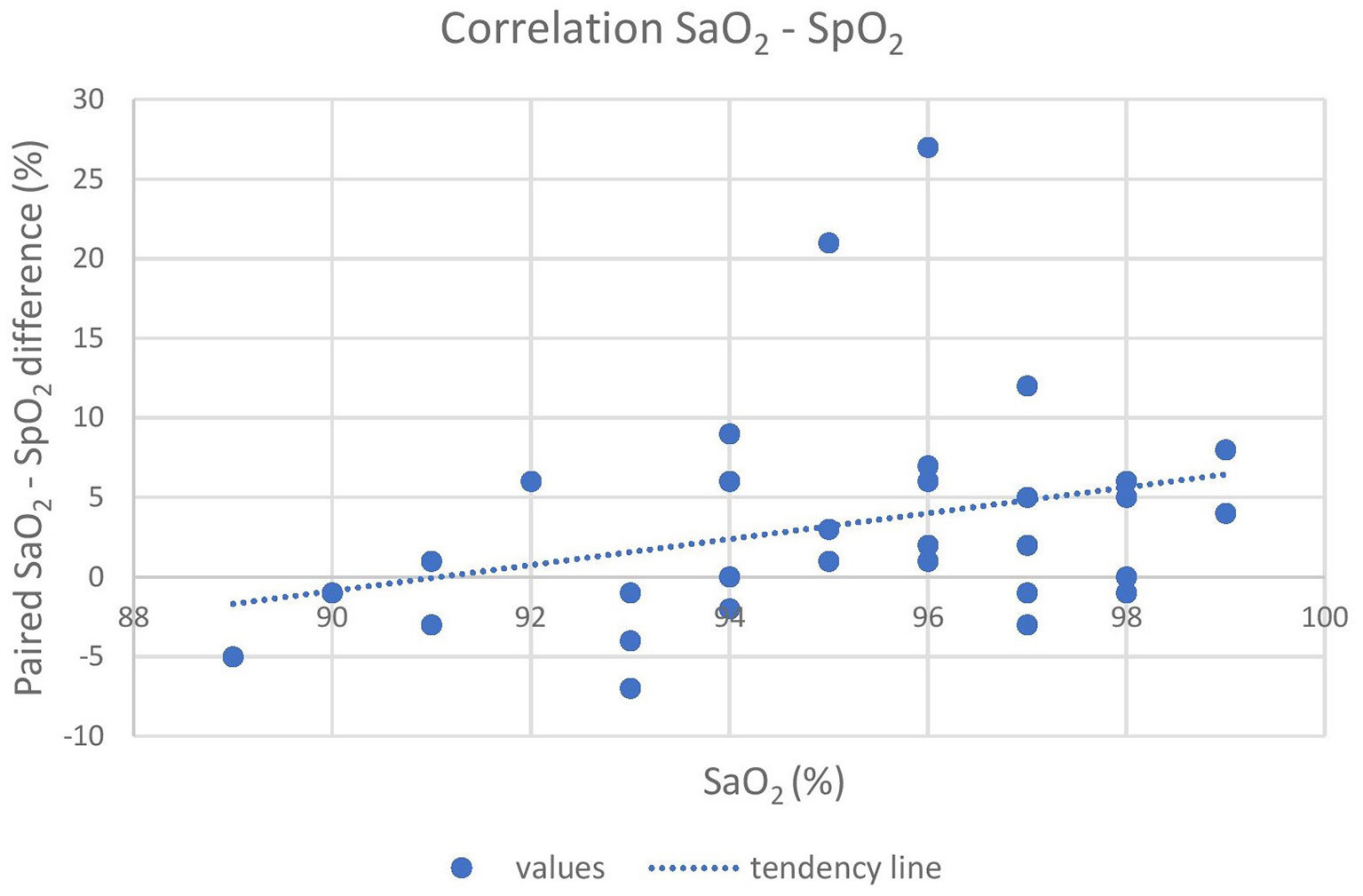
The graph shows the poor correlation between the pulse-oximeter and the iSTAT (X-axis) results. The difference between SaO_2_, and SpO_2_ is reported on the Y-axis. A tendency of the pulse-oximeter to underestimate actual SaO_2_, especially at high readings, is shown. SaO_2_, arterial oxyhemoglobin saturation; SpO_2_, peripheral oxyhemoglobin saturation.

Oxygen was delivered in only 17 out of 21 animals due to the occurrence of multiple trapped and immobilized animals at a time and the availability of only one portable oxygen concentrator. Despite the pulse amplitude and quality detected by the pulse oximeter were subjectively good, there was no acceptable correlation between pulse-oximetry values for SpO_2_ and blood gas analysis for SaO_2_ (|ρ_c_| < 0.2). Agreement was below both the statistical and clinical thresholds, since only 93.7 and 65.6% of the values lied within the LoA and the CAL intervals, respectively, in the Bland-Altman plot. Pulse-oximetry underestimated blood oxygenation in 63% of cases regardless of the group and to a higher extent for higher SpO_2_ values. Concordance, correlation and agreement between SpO_2_ and SaO_2_ measurements are presented in Figures 1–3.

Mean Tr, HR and RR trends were significantly different among protocol groups. Average Tr was higher (*p* = 0.005) in wild boars in the MKB group compared to the MTZ group, with a decreasing trend over time in both groups. Hyperthermia occurred in 3 out of 7 boars in the MK group and in 3 out of 6 boars in the MKB group. Hyperthermia was seen only in 1 out of 8 animals in the MTZ group. Mean HR was higher (*p* = 0.048) in the MK group compared to the other groups, remaining constant or decreasing over time in the MTZ and MK groups. RR was increasing in all animals, with significantly lower mean values (*p* = 0.042) in the MK group compared to the MTZ group. Details of Tr, HR and RR are reported in Table 2.

**Table 2:** Physiological variables of cage-trapped wild boars anesthetized using medetomidine-tiletamine-zolazepam (MTZ; *n* = 8) or medetomidine-ketamine (MK; *n* = 7) or medetomidine-ketamine-butorphanol (MKB; *n* = 6).

			**Tr**				**HR**				**RR**			
**Group**		**Delta T0**	**T0**	**T1**	**T2**	**T3**	**T0**	**T1**	**T2**	**T3**	**T0**	**T1**	**T2**	**T3**
MTZ	Mean	0:20	38.6	38.5	38.1	37.6	65	58	58	58	36	37	40	47
	± SD	0:11	0.6	0.8	1.0	0.5	8	4	9	10	12	9	11	11
	*n*	8	8	6	7	5	7	6	7	5	7	6	7	4
MK	Mean	0:17	39.0	39.2	38.8	39.2	68	63	61	61	24	24	32	31
	± SD	0:07	0.8	0.9	0.9	0.7	9	9	6	9	11	12	17	10
	*n*	7	7	7	6	3	7	7	6	3	7	7	6	3
MKB	Mean	0:26	39.6	39.4	39.4	39.3	56	56	57	61	25	36	37	45
	± SD	0:07	0.9	1.0	1.0	0.2	17	17	14	13	11	15	15	4
	*n*	6	6	6	6	2	6	6	6	2	6	6	6	2

*Time from first pole-syringe injection to the start of the monitoring (Delta T0) and rectal temperature (Tr), heart rate (HR), and respiratory rate (RR) at t0 and after 10, 20 and 30 min, in 21 anesthetized wild boars*.

### Arterial Blood Gas Analysis

Twenty out of 21 anesthetized wild boars were sampled for blood gas, electrolyte, and hematological analysis (8 from the MTZ group and 6 each for the MK and MKB groups). For two of those animals (MK and MKB groups) we were not able to perform an arterial sample within the timeframe. Three individuals (1 from the MTZ group and 2 from the MKB group) were excluded from the blood gas analysis due to artifacts in one of the two arterial samples (i.e., mixed venous/arterial blood sample or prolonged exposure to ambient air). The comparison between the first and the second arterial sample was conducted only for those individuals which received oxygen supplementation. Hypoxemia occurred regardless of the group, although with differences in frequency and extent. All wild boars in the MKB group developed mild hypoxemia, except one individual which developed moderate hypoxemia (69 mmHg). Mild hypoxemia occurred in three out of seven animals in the MTZ group, and in one out of six individuals in the MK group. The alveolar-arterial oxygen tension difference was increased in all groups, with higher values (23 vs. 19 vs. 19 mmHg) in the MKB group. Acidemia and mild hypercapnia were seen in all groups. Lactate was below 6 mmol/L in all wild boars, with no significant differences among the groups. At the time of the second arterial sample, Tr was significantly higher in the MKB group than in MTZ group and K^2+^ was significantly higher in the MKB group compared to the MTZ and MK groups. Details of the complications occurred during the immobilizations are reported in Table 3.

**Table 3:** Complications during the anesthesia of cage-trapped wild boars anesthetized using medetomidine-tiletamine-zolazepam (MTZ; *n* = 8) or medetomidine-ketamine (MK; *n* = 7) or medetomidine-ketamine-butorphanol (MKB; *n* = 6).

	**Induction problems**		**Hyperthermia**	**Hypoxemia**		**Hypercapnia**	**Acidemia**
**Group**	**RD**	**ID**		**Mild**	**Moderate**		
**MTZ**	2 (8)	2 (8)	1 (8)	3 (7)	0 (7)	5 (7)	8 (8)
**MK**	1 (7)	1 (7)	3 (7)	1 (6)	0 (6)	5 (6)	6 (6)
**MKB**	0 (6)	4 (6)	3 (6)	3 (4)	1 (4)	4 (4)	5 (6)

*Complications during the anesthesia of 21 wild boars. Both the number of the individuals that presented each complication and the total number of individuals (in brackets) of each group are reported. Induction problems are defined as animal not being recumbent after 10 min (recumbency delay, RD) or not completely immobilized 20 min post injection (induction delay, ID). Hyperthermia is defined as at least two consecutive temperature measurements above 39.5°C. Hypoxemia is defined as mild (PaO_2_ 80–70 mmHg; SaO_2_ 97–90%) or moderate (PaO_2_ 69–60; SaO_2_ 89–78%) according to the iSTAT® PaO_2_ and SaO_2_. Hypercapnia and acidemia are based on reference intervals of PaCO_2_ and pH from unanesthetized wild boars (21–25) and domestic pigs (26–30)*.

No statistically significant changes in any blood variable in the three groups were identified after the oxygen delivery period. Also, pH, lactate, ${\text{HCO}}_{3}^{-}$, and especially PaO_2_ and SaO_2_ improved during oxygen supplementation. Hypoxemia and hypercapnia improved by the time of the second sample also in animals that did not receive supplemental oxygen. At the time of the second sample, the mean PaO_2_ and PaCO_2_ were 117 and 52 mmHg in animals receiving supplemental oxygen and 99 and 48 mmHg in the ones who did not receive any supplementation. Details of the measured variables related to blood gas analysis, hematology and serum biochemistry are presented in Table 4.

**Table 4A:** Blood gas, hematological and serum biochemistry variables before oxygen supplementation of cage-trapped wild boars anesthetized using medetomidine-tiletamine-zolazepam (MTZ; *n* = 8) or medetomidine-ketamine (MK; *n* = 6) or medetomidine-ketamine-butorphanol (MKB; *n* = 6).

**A**	**Group**	**MTZ**				**MK**				**MKB**				
		***n***	**Mean**	**SD**	**Range**	***n***	**Mean**	**SD**	**Range**	***n***	**Mean**	**SD**	**Range**	**Reference range**
1° sample	pH	8	7.374	0.042	7.286–7.430	6	7.393	0.037	7.351–7.444	6	7.385	0.055	7.329–7.473	7.453–7.540
	PaCO_2_ (mmHg)	7	50.2	6.8	41.6–58.5	6	49.4	5.5	41.0–55.8	4	56.3	2.7	53.9–60.1	35.8–44.4
	PaO_2_ (mmHg)	7	84.6	9.0	85.0–99.0	6	85.5	6.5	77.0–95.0	4	74.8	5.0	69.0–79.0	81.6–107.7
	BE (mEq/L)	8	3.0	2.4	0–7.0	6	5	5.2	−1.0–10.0	6	6	2.2	3.0–8.0	4.6–12.6
	${\text{HCO}}_{3}^{-}$ (mEq/L)	8	28.8	2.6	24.9–35.0	6	29.7	5.1	23.9–35.3	6	30.7	2.5	28.5–33.1	27.8–36.6
	TCO_2_ (mEq/L)	7	29.9	2.8	26.0–35.0	6	31.2	5.4	25.0–37.0	4	32.3	2.6	30.0–35.0	28.9–38.0
	SaO_2_ (%)	7	94	2	91–97	6	95	1	93–97	4	91	2	89–94	97–100
	SpO_2_ (%)	7	93	3	90–98	6	89	9	74–97	4	91	4	86–94	95–100
	PAO_2_ (mmHg)	7	104	6	96–112	6	104	5	99–112	4	98	3	94–99	108.7–116.6^*^
	P_(A-a)_O_2_ (mmHg)	7	19	4	13–27	6	19	4	12–22	4	23	3	20–27	<15
	Lactate (mmol/L)	8	2.7	1.4	1.5–5.8	6	2.9	0.9	1.4–3.9	6	2.4	1.0	1.3–4.1	4.6–36.7
	Tr (°C)	8	38.7	0.7	37.4–39.6	6	38.9	0.8	37.9–40.1	6	39.4	0.9	38.1–40.6	38.7–39.5
	Na (mmol/L)	7	146	4	141–152	6	147	3	144–153	6	149	3	144–153	126–157
	K (mmol/L)	7	3.6	0.2	3.4–3.9	6	3.4	0.3	3.0–3.7	5	4.1	0.6	3.6–5.0	2.5–13.5
	Cl (mmol/L)	0	–	–	–	0	–	–	–	4	109	3	106–112	89–108
	AnGap (mmol/L)	0	–	–	–	0	–	–	–	4	13.5	2.6	10.0–14.8	10.0–25.0
	iCa (mmol/L)	7	1.26	0.15	1.04–1.51	6	1.23	0.06	1.17–1.31	1	1.25	0.00	1.25	1.38–2.90
	BUN (mg/dl)	0	–	–	–	0	–	–	–	4	14.2	2.6	12.0–18.0	3.3–16.7
	Glucose (mg/dl)	7	178	47	113–233	6	194	38	161–261	5	247	48	199–313	90–350
	Hct (%)	7	36.1	3.0	34.0–41.0	6	37.0	3.7	32.0–42.0	5	38.2	3.9	33.0–43.0	29.0–53.0
	Hb (g/dl)	7	12.3	1.0	11.6–13.9	6	12.6	1.3	10.9–14.3	5	13.0	1.3	11.2–14.6	10.1–17.8

* *Based on the P_B_ at the moment of the captures and on reference intervals for PaCO_2_*.

**Table 4B:** Blood gas, hematological and serum biochemistry variables after oxygen supplementation in 20 anesthetized wild boars.

**B**	**Group**	**MTZ**				**MK**				**MKB**				
		***n***	**Mean**	**SD**	**Range**	***n***	**Mean**	**SD**	**Range**	***n***	**Mean**	**SD**	**Range**	**Reference range**
2° sample	pH	8	7.397	0.035	7.352–7.445	5	7.439	0.075	7.352–7.534	5	7.386	0.024	7.345–7.405	7.453–7.540
	PaCO_2_ (mmHg)	8	49.6	5.4	41.8–57.8	4	50.8	5.9	43.5–56.6	5	53.8	3.7	49.8–56.4	35.8–44.4
	PaO_2_ (mmHg)	8	98.0	13.3	84.0–120.0	4	112.8	14.7	102.1–134.8	5	111.0	28.2	74.5–144.6	81.6–107.7
	BE (mEq/L)	8	5.0	2.2	1.0–8.0	5	8.2	5.3	1.0–12.0	5	6.1	2.1	4.0–9.0	4.6–12.6
	${\text{HCO}}_{3}^{-}$ (mEq/L)	8	30.2	2.7	25.0–33.7	5	32.6	5.1	25.7–38.0	5	31.6	2.3	29.3–34.1	27.8–36.6
	TCO_2_ (mEq/L)	8	31.6	2.8	26.0–35.0	4	34.0	6.1	27.0–40.0	5	33.2	2.6	31.0–36.0	28.9–38.0
	SaO_2_ (%)	8	97	2	93–98	4	98	1	97–99	5	96	3	91–99	97–100
	SpO_2_ (%)	8	94	5	85–100	4	94	4	91–100	3	86	15	69–95	95–100
	Lactate (mmol/L)	8	2.0	1.2	0.9–4.6	5	1.8	0.7	1.1–2.9	5	2.0	0.9	1.2–3.5	4.6–36.7
	Tr (°C)	8	37.9	0.9	37.1–39.5	5	38.6	0.8	37.8–39.9	5	39.3	0.8	38.1–40.2	38.7–39.5
	Na (mmol/L)	8	145	3	141–149	5	148	3	143–152	5	148	3	144–152	126–157
	K (mmol/L)	8	3.8	0.3	3.3–4.3	5	3.8	0.3	3.3–4.0	5	4.3	0.2	4.0–4.5	2.5–13.5
	Cl (mmol/L)	0	–	–	–	0	–	–	–	4	110	6	105–117	89–108
	AnGap (mmol/L)	0	–	–	–	0	–	–	–	4	10.7	3.3	9.0–14.0	10.0–25.0
	iCa (mmol/L)	8	1.26	0.13	1.06–1.50	5	1.23	0.05	1.15–1.28	1	1.26	0.00	1.26	1.38–2.90
	BUN (mg/dl)	0	–	–	–	0	–	–	–	4	13	1.6	11.0–15.0	3.3–16.7
	Glucose (mg/dl)	8	229	85	112–350	5	227	33	192–275	5	262	42	207–321	90–350
	Hct (%)	8	34.4	2.6	32.0–38.0	5	34.6	4.0	29.0–39.0	5	37.4	5.3	32.0–46.0	29.0–53.0
	Hb (g/dl)	8	11.7	0.9	10.9–12.9	5	11.8	1.4	9.9–13.3	5	12.7	1.8	10.9–15.6	10.1–17.8

*Mean, SD and range values for blood gas, hematological and serum variables in 20 anesthetized wild boars (Sus scrofa). Results of both 1° and 2° samples (15 min apart while supplementing oxygen) are reported. Reference intervals from unanesthetized wild boars (21–25) and domestic pigs (26–30)*.

*PaCO_2_, arterial partial pressure of carbon dioxide; PaO_2_, arterial partial pressure of oxygen; BE, base excess; ${HCO}_{\text{3}}^{-}$, bicarbonate; TCO_2_, total carbon dioxide; SaO_2_, arterial oxyhemoglobin saturation; SpO_2_, peripheral oxyhemoglobin saturation; PAO_2_, alveolar partial pressure of oxygen; P_(A−a)_O_2_, alveolar-arterial oxygen tension difference; Tr, rectal temperature; Na, sodium; K, potassium; Cl, chloride, AnGap, anion gap, iCa, ionized calcium; BUN, urea; Hct, hematocrit; Hb, hemoglobin*.

## Discussion

### Efficiency and Reliability of the Capture

Short inductions must be pursued when immobilizing wild ungulates, since animals anesthetized with only one injection present a lower risk of developing stress, hyperthermia, tachycardia, and slower recoveries (6). Although the drug protocols evaluated in this study did not result in significantly different induction times, the anesthetic efficiency (percentage of immobilized wild boars with a single injection) was lower than previously reported with the same drug combinations (18, 19, 31). In fact, a second injection was often necessary, particularly in the MKB group. This difference is possibly due to the large volume of the MKB combination (>6 ml), which may have led to delayed absorption of the drugs. Another reason for prolonged induction times could be an incorrect drug placement into the subcutaneous fat, although efficacy and onset between subcutaneous and IM injections were not significantly different in similar studies in domestic pigs (32). Furthermore, studies on wild boars found a direct relationship between the number of animals captured in the same trap and the required drug dose to achieve complete immobilization, due to the high levels of stress of the captured animals (33). Thus, the event of more than one animal caught in the same trap could have led to delayed induction, although it occurred only in three captures.

The majority of the induction problems occurred in MKB and MTZ (when the planned dose was administered) groups, whereas animals from MK group underwent a smoother induction. The hypothesis of an inadequate dose of ketamine and tiletamine-zolazepam leading to induction problems is supported by previous studies in wild boars and domestic pigs in which higher doses were adopted, achieving a relatively rapid induction (4, 18, 34). However, the depth of immobilization for boars injected with ketamine-based protocols was lighter, and a second dose of anesthetic was required after 20–40 min in two cases in each group to carry out further procedures. This reflects the shorter half-life of ketamine compared to tiletamine-zolazepam.

The administration of extra doses of ketamine, especially in the MK group, likely contributed to the difference in recovery time among groups. Nonetheless, the time of achievement of coordination after atipamezole administration was longer than previously reported in all three groups (17, 19). The addition of butorphanol at the present dose might have masked the effects of ketamine in MKB group, consequently reducing the duration of the recovery time compared to MK protocol, as previously documented (18). However, butorphanol did not elicit any clear improvement on the depth of the anesthesia, contrarily to previous findings (4), probably due to the lower dose of medetomidine given in MKB group. In the light of similar studies in suids, we can speculate that quality and reliability of the induction with ketamine-based combinations heavily depends on the ratio between the doses of the dissociative anesthetic and the sedatives (35).

Ataxia and rough, long recoveries occurred after atipamezole administration regardless of the drug protocol. This is consistent with other studies on cyclohexamine anesthesia in this species (1, 4, 7, 34) and it could be attributed to the effects of the active metabolites of ketamine (i.e., norketamine), whose half-life is considerably longer than ketamine in humans (36) and in domestic animals (37). However, the species-specific pharmacokinetics of ketamine and its metabolites is not completely understood in the wild boar yet, leaving such hypothesis at the speculative level. Although tiletamine seems not to be converted in active substances in pigs, its duration of action is more extended than ketamine (38), leading to similar effects. Adding butorphanol to the MK protocol did not clearly improve the quality of the recovery after reversal. The effects of butorphanol on the recovery have been debated in a number of studies on wild boars and domestic pigs (4, 18, 19), with no univocal answer.

It is important to consider that the differences in both age and average body mass among groups may have influenced the variables related to capture and immobilization. In fact the initial dose was increased by 150% in MTZ, since induction problems were noticed. Although neither sex nor age has been confirmed to affect the required doses and induction in wild boars to date, the physiologic responses in this study must be interpreted carefully. Nevertheless, no difference which could be elicited by such bias occurred in the present study.

The pre-release mortality reported here (6.2%) falls within the previously reported 1.6–10.6% range for this species (1, 33), with no fatalities occurring during the anesthesia in the present study.

### Physiological Variables

Hyperthermia occurred most frequently in MK and MKB groups. A higher Tr associated with a longer induction time in the MKB group than in the MTZ group is consistent with the findings of a pilot study by one of the authors (Arnemo, unpublished data).

In wild boars and in domestic pigs, marked bradycardia and transient pulmonary and systemic hypertension have been reported when using medetomidine as sedative (8, 9, 19, 34). In the MKB protocol, butorphanol was likely to further contribute to the bradycardia due to its confirmed cardiodepressant properties in mammals (39). Nevertheless, both ketamine and tiletamine elicit an increase in HR when added to the anesthetic protocol, easing such profound bradycardia, as reported in similar studies (4).

The RR was approximately ~50% lower than reported in other studies on wild boars and domestic pigs (8, 19), although similar values are reported in warthogs (*Phacochoerus africanus*) immobilized with similar combinations (31). Contrarily to what is reported in literature for pigs undergoing ketamine administration (10, 35), no wild boar in the current study developed tachypnea once fully sedated. In fact, boars anesthetized with MK showed a lower RR compared to the other groups. A drop of both Tr and HR, and an increase of RR over time frequently occurs in suids immobilized with the same drug protocols (8, 19, 34).

In this study, we corroborate previous findings (12, 13, 40) where the agreement between SaO_2_ (iSTAT) and SpO_2_ (pulse oximeter) was poor, particularly at low values of SpO_2_ (Figures 1, 2). This could be due to several factors, including species-specific differences and to the use of alpha-2 adrenoceptor agonist drugs (41). Although the SaO_2_ underestimation at high values that resulted in this study is clinically safer than its overestimation, researchers should also take into account other indicators (i.e., color of mucus membranes and changes in respiratory rate and tidal volume) when assessing hypoxemia in this species. Underestimation at high saturation levels is consistent in brown bears (*Ursus arctos*) (14), musk oxen (*Ovibos moschatus*) (13) and white-tailed deer (*Odocoileus virginianus*) (12) and is associated with overestimation at low levels. Further, although low in accuracy, the pulse oximeter can be reliably used to monitor the trend of SpO_2_ over time during the immobilization.

### Arterial Blood Gas Analysis

Simple acute respiratory acidosis occurred in all three groups in the current study. This is the most common acid-base disorder during anesthesia in spontaneous ventilation (42), with hypercapnia and hypoxemia, usually due to hypoventilation caused by a decrease in respiratory rate and/or tidal volume. This disorder has been reported for all three protocols when used in domestic pigs (4, 34), and it was especially prevalent in boars anesthetized with MKB in the current study. The boars immobilized with MKB also presented higher P_(A−a)_O_2_ compared to the other groups. A number of studies on animals immobilized with similar protocols confirm that ventilation-alveolar perfusion mismatch is another remarkable cause of hypoxemia, mainly due to alpha-2-agonist-induced pulmonary vasoconstriction and prolonged recumbency (43, 44), and would be consistent with the increased P_(A−a)_O_2_ of the wild boars in this study, regardless of the group. Additionally, the degree of medetomidine-induced increase in pulmonary resistance is considered to be higher in suids than in carnivores (19, 45), and is more severe when butorphanol is added to the combination (17, 46). The mild non-significant differences in the current study among protocol groups, are consistent with previous findings in pigs anesthetized with MKB, as this protocol is reported to elicit a remarkable depressant effect on blood gas and acid-base status (5, 19) compared to MTZ (18) or MK (4). The hypoxia detected in the wild boars at the time of the first arterial blood sample was only mild (and moderate in one individual), milder than in several other species undergoing anesthesia in the field, although different drug protocols and capture methods were used (14). In addition to this, the supplemental oxygen flow was deliberately very conservative considering the size of the immobilized animals (47) due to the mild grade of the hypoxemia encountered. A more pronounced increase of PaO_2_ (~172 mmHg) is reported in anesthetized pigs in spontaneous ventilation with a FiO_2_ of 0.5 and an intratracheal oxygen flow of 30–50 ml/kg/min (42). Being the oxygen flow not intratracheal and only about 5–20 ml/kg/min in this study, it might have not produced a clinically significant increase in P_A_O_2_. The cardiopulmonary effects of prolonged lateral recumbency, the presence of right-to-left intrapulmonary shunting and diffusion impairment could not be determined in this study. All these factors might have contributed to the fact that no significant change in PaO_2_ nor SaO_2_ were detected following oxygen supplementation.

Although the wild boars did not experience hypoxemia of clinically concerning levels in any group, oxygen supplementation resolved the mild to moderate hypoxemia in all wild boars in the present study, hence we recommend it for all three combinations assessed here. Oxygen has been previously recommended for the MKB protocol (5, 17). Although most animals were still acidemic after oxygen supplementation, the pH slightly improved in the MTZ and to a higher extent in MK group despite the significantly lower RR and the sustained hypercapnia. This might be explained by the marked decrease in lactate over time in the wild boars anesthetized with MK.

Both the respiratory acidosis and the hypercapnia occurred following the administration of the drug combinations in this study were mild and of limited clinical impact compared to other studies in anesthetized wildlife (12–14). This reflects the safety of such capture and drug combinations in regards to the respiratory and metabolic status in wild boars. In spontaneous ventilation circumstances, hypercapnia, and thus respiratory acidosis, resolves only following a change in minute ventilation (RR and/or tidal volume). The fact that the average levels of PaCO_2_ did not change significantly over time indirectly suggests that minute ventilation is likely to remain stable in animals immobilized with these drug combinations.

Bicarbonates and BE increased slightly over time, this is perfectly predictable as the metabolic compensation usually begins within 10 min from the start of the acute respiratory acidosis (19, 48).

### Limits of the Research

The exiguous number of the animals anesthetized with the MTZ, the MK and the MKB drug combinations leads to significant statistical limitations in this study, risking not to detect significant differences in all measured variables. Extending the research to a larger sample and including a larger control sample of wild boars not receiving supplemental oxygen would result in a more reliable interpretation and a more solid inference to a wider population. Additionally, the results in this study are highly dependent of the doses of the anesthetic combinations used, therefore they should be interpreted carefully as different dose ratios of the drug combinations may have resulted in different outcomes.

Wildlife capture inevitably leads to heterogeneity in regards to age, body mass, sex and number of individuals in each group. A more standardized study sample would decrease the risks of statistical bias. The implementation of a standard scoring system to assess the anesthetic depth would have increased rigor and statistical relevance, helping to draw more objective conclusions on the quality of the anesthesia.

Although portable analyzers such as i-STAT® are the most accurate instrument available in field settings to measure blood gases, they present some limitations compared to benchtop analyzers that are considered to be the gold-standard. Further, i-STAT® calculates SaO_2_ following algorithms based on a pre-set oxyhemoglobin saturation curve, which is species-specific. Hence a validation of the accuracy of i-STAT® for blood gas variables in wild boars would be recommendable.

As the bar graph on the pulse oximeter display evaluating the pulse amplitude is assessed subjectively by the reader, further inaccuracy due to anomalies in the probe-oxyhemoglobin interface (e.g., unsuitable probe site, medetomidine-induced vasoconstriction, movements, etc.) could not be always ruled out.

## Conclusions

Evaluating both advantages and disadvantages of the three anesthetic protocols, we have demonstrated that ketamine-based protocols were reliable to safely immobilize wild boars to carry out short procedures. The choice of the drug protocol depends on the purpose and the expected duration of the procedures, making MK and MKB combinations effective and reliable for procedures shorter than 40 min. After that time, a supplemental dose of ketamine may be required. Regarding the choice between these two protocols, it is important to consider their impact on induction time, arterial blood gases, pH and recovery time. Despite all three protocols combined with the capture method in this study elicited minimal impact on the respiratory function, we strongly recommend the use of supplemental oxygen to prevent and treat hypoxemia in wild boars, especially if the MKB protocol is used at the reported doses. Ataxia and rough recoveries seem to be a common occurrence in wild boar anesthesia, regardless of the drug protocol adopted. In case of longer procedures, MTZ combination at the increased dose would be preferred for wild boar anesthesia, due to its milder cardiopulmonary and hematological effects.

The pulse-oximeter readings did not correlate with the arterial oxyhemoglobin saturation measured with iSTAT®, underestimating the real values in most cases and to a higher extent at higher readings. Further studies are recommended on the species-specific hemoglobin dissociation curve and the accuracy of pulse-oximeters in wild boars immobilized with similar protocols. Due to the limitations of the present study, further research with a higher number of wild boars anesthetized with the same drug combinations is needed to corroborate the findings and to extend the conclusion to the population level. Additional research should assess whether the combination of the same drugs, but with different doses, would elicit a lower incidence of induction problems and prevent the requirement for an additional anesthetic dose.

## Data Availability Statement

The original contributions presented in the study are included in the article/supplementary material, further inquiries can be directed to the corresponding author/s.

## Ethics Statement

The animal study was reviewed and approved by SR participated to a training course provided by the French National Museum of Natural History (Muséum National d'Histoire Naturelle, MNHN) and the French National Centre for Scientific Research (Centre National de la Recherche Scientifique, CNRS), in collaboration with the French Office for Biodiversity (Office Français pour la Biodiversité, OFB). At the end of the course, SR passed successfully the exam and was awarded an official permit for capture, anesthesia, handling, sample collection and any other use of the animals for scientific purposes.

## Author Contributions

The study was conceived and designed by SR, JA, and AE. SR planned, organized and led the captures. SR, BF, AE, SK, and DB carried out the field sampling and data collection. Data was prepared by BF, AE, DB, and JM. JM analyzed the data and drafted the manuscript. SR and JM contributed to interpretation of the results. All authors contributed to the article and approved the submitted version.

## Conflict of Interest

The authors declare that the research was conducted in the absence of any commercial or financial relationships that could be construed as a potential conflict of interest.

## Abbreviations

MTZ: medetomidine-tiletamine-zolazepam
MK: medetomidine-ketamine
MKB: medetomidine-ketamine-butorphanol
Tr: rectal temperature
HR: heart rate
SpO_2_: peripheral oxyhemoglobin saturation (pulse-oximeter)
RR: respiratory rate
PaO_2_: arterial partial pressure of oxygen
PaCO_2_: arterial partial pressure of carbon dioxide
SaO_2_: arterial oxyhemoglobin saturation (iSTAT®)
LoA: Limit of Agreement
CAL: Clinically Acceptable Limit.
