# Erratum: Evaluation of Three Medetomidine-Based Anesthetic Protocols in Free-Ranging Wild Boars (*Sus scrofa*)

**DOI:** 10.3389/fvets.2021.693925

**Published:** 2021-04-28

**Authors:** Frontiers Production Office

**Affiliations:** Frontiers Media SA, Lausanne, Switzerland

**Keywords:** butorphanol, blood gas, oxygen, anesthesia, capture, medetomidine, *Sus scrofa*, wild boar (*Sus scrofa* L.)

Due to a production error, there was a mistake in the captions of **Figure 1**, **Figure 2**, and **Figure 3** as published. The corrected figure captions appear below.

**Figure 1**. Bland-Altman analysis plots the difference between concurrent SaO_2_ and SpO_2_ values (Y-axis) against the mean between concurrent SaO_2_ and SpO_2_ values (X-axis). Mean difference (bias), upper LoA (bias + 1.96 SD), and lower LoA (bias – 1.96 SD) between paired values are represented with straight lines. Upper CAL (5%) and lower CAL (−5%) are represented with dashed lines. LoA, Limit of Agreement; CAL, Clinically Acceptable Limit; SaO_2_, arterial oxyhemoglobin saturation; SpO_2_, peripheral oxyhemoglobin saturation.

**Figure 2**. The graph shows the poor concordance between the pulse-oximeter (Y-axis) and the iSTAT (X-axis) results. The blue line represents the equality line, that is for the absolute concordance. SaO_2_, arterial oxyhemoglobin saturation; SpO_2_, peripheral oxyhemoglobin saturation.

**Figure 3**. The graph shows the poor correlation between the pulse-oximeter and the iSTAT (X-axis) results. The difference between SaO_2_, and SpO_2_ is reported on the Y-axis. A tendency of the pulse-oximeter to underestimate actual SaO_2_, especially at high readings, is shown. SaO_2_, arterial oxyhemoglobin saturation; SpO_2_, peripheral oxyhemoglobin saturation.

The publisher apologizes for these mistakes. The original article has been updated.

